# Establishment of a urogynecology cadaver-based hands-on workshop

**DOI:** 10.4314/ahs.v23i1.64

**Published:** 2023-03

**Authors:** Hassan M Elbiss, Fikri M Abu-Zidan

**Affiliations:** 1 Department of Obstetrics and Gynaecology, College of Medicine and Health Sciences UAE University, Al-Ain, United Arab Emirates; 2 The Research Office, College of Medicine and Health Sciences UAE University, Al-Ain, United Arab Emirates

**Keywords:** Cadaver, pelvic floor, surgery, skills, training, assessment

## Abstract

**Background:**

It is important to train gynecologists and urologists on the principles of urogynecological surgery.

**Objectives:**

To report our recent experience in developing a curriculum, application, and the learning outcome of a urogynecology workshop using cadaver training.

**Methods:**

A full day structured urogynecology cadaver-based educational hands-on course was developed. The theoretical component consisted of lectures on female urinary incontinence and genital prolapse. Hands-on training was on three cadaver stations: First to instruct and guide through the surgical steps of transobturator vaginal tape; second to perform sacrospinous fixation; and third to insert of vaginal mesh. Knowledge gained was evaluated using multiple choice questions. The course was evaluated using a structured seven-point Likert type questionnaire.

**Results:**

There was an a statistically significant improvement in the post-test MCQ marks compared with the pre-test marks (P <0.01) and in the pass percentage (7% compared with 100%, P< 0.001). The overall rating of the workshop was 6.7 out of 7.

**Conclusions:**

Our course was highly valued by the participants who came from all over the Middle East. The course was enjoyable and achieved its objectives while the participants gained new knowledge and surgical skills.

## Introduction

Urogynecology, a subspecialty within obstetrics and gynecology, focuses on disorders of the female pelvic floor. Symptoms of pelvic floor prolapse, and female urinary incontinence are common among women 1,2, which are best treated by less invasive and effective novel urogynecology surgery. It is important to train gynecologists and urologists on the principles of urogynecological surgery to achieve the theoretical knowledge and practical skills that can be successfully applied in their clinical practice. This should include different levels of skills for urogynecological procedures and how to deal with the intraoperative and postoperative complications.

Mannequins and training boxes are used to train surgeons of different specialties in performing new surgical skills^3^,^4^. Cadaver models are ideal and close to real life for urogynecology and urology training. These models improve knowledge on pelvic anatomy and stereotactic skills needed for gynecological procedures 5,6. Their use is restricted due to ethical, cultural or belief considerations. It is important to evaluate the educational value of cadaver training for gynecologists and urologists at different skill levels needed for urogynaecological surgery. We aim to report our recent experience in developing a curriculum, application, and the learning outcome of a urogynecology workshop using cadaver-based training.

## Methods

A full day urogynecology cadaver workshop was held during December 2016 at University of Sharjah Clinical Training Center, United Arab Emirates. The workshop was announced as part of the 5^th^ Emirates International Urological Conference 2016 and was sponsored by the Coloplast Company, Dubai, UAE.

Seventeen participants from the Middle east and North Africa having different specialties and levels of experience attended the workshop. Ten were urologists, five gynecologist and two obstetricians and gynecologists. Eleven participants were consultants, and six specialists. The theoretical component of the course was held in the morning. Its content is shown in Table 1. It consisted of several lectures covering topics on female urinary incontinence and genital prolapse.

**Table 1:** The theoretical content of the course

Subject	Time (min)	Content
1. Introduction	10	Objectives and general overview of the course
2. Epidemiology and pathophysiology of female stress urinary incontinence (USI)	20	Overall prevalence of female USI regionally and internationally and description of possible mechanisms of developing of USI.
3. Surgical anatomy of SUI	20	Explaining the surgical anatomy relevant to sub-urethral sling.
4. Which sling is the best for which patient?	20	Discuss various types of suburethral slings and justify which one is best for individual patient.
5. SUI complications and management	30	Explaining the complications of SUI surgery and how to avoid and manage them.
6. Tips and tricks of sub-urethral slings	30	Explaining the good clinical practice in performing SUI surgery.
7. Sacrospinous fixation (SSF) for uterine and vaginal vault prolapses	30	Explaining how to select patients for SSF and understanding the anatomy relevant to this surgery
8. Vaginal mesh implant for uterovaginal prolapse	30	Explaining how to select patients for vaginal mesh implant and understanding recent warning regarding complications of vaginal mesh surgeries.

**Figure 1 (A-B):**
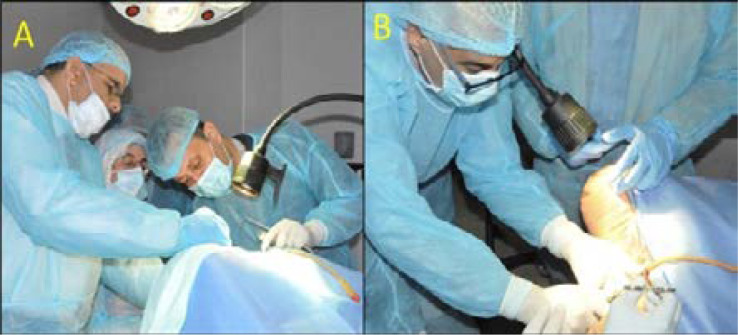
Practical sessions were held on soft tissue preserved cadavers under direct supervision of urogynecology expert surgeons.

The Practical sessions were held in the afternoon on three soft tissue preserved cadavers. (Figure 1). There were three stations to learn different operative skills (Table 2)

**Table 2:** The practical content of the course

Practical station	Objectives	Task
Transobturator (TOT) vaginal tape	To allow participants to have good understanding of relevant anatomical landmarks and how to be able to insert TOT	Demonstrate then let participants perform the procedure under direct supervision
Retropubic Sling System	To learn how to perform retropubic sling from top-down	Demonstrate then let participants perform procedure under direct supervision
Sacrospinous fixation	To learn how to dissect the vagina in order to identify ischial spines then uterosacral ligaments	Applying correctly sutures to sacrospinous ligaments
Vaginal mesh surgery	To learn how to dissect the vagina to insert vaginal mesh and to understand the pelvic landmarks for the specific mesh	Insert specific vaginal mesh under direct supervision

Each participant rotated around these three stations to practice each skill. Depending on the learning needs and individual interest, some participants spent more time at some stations. This was intended so that the curriculum offers a flexible and tailored course that matches the requirements of each participant.

Harden et al^7^ proposed an educational curriculum based on the SPICES approach. The workshop's curriculum used the recommended approaches of being 1) Student-centered which is tailored towards the needs of the participants, 2) Problem-based by discussing clinical problems, 3) Integrated by integrating both basic and clinical sciences, 4) Elective in which the participants selected their learning needs, and finally the 5) Systematic approach in which all participants received the same learning experience. The only missing approach was the Community-based approach. In addition, the curriculum covers the whole spectrum of the Miller's Pyramid of clinical competencies which includes knowledge (Knows, and knows how) and action (shows how ands does) 8, 9.

The participants provided baseline information regarding their gender, medical degree, country of origin, job title and attendance at previous relevant courses. The participants answered 20 multiple choice questions (MCQs) before and after completion of the course which were developed to cover the course content having face validity. Each item had 5 answer options. At the end of the course, participants responded anonymously to a structured questionnaire to evaluate the course. The questionnaire consisted of 14 statements and 3 open comments covering different aspects of the course. The participants answered each question on a seven-point Likert type scale (1 very poor, 2 poor, 3 fair, 4 acceptable, 5 good, 6 very good, 7 outstanding).

Data were presented as median (range) and mean (SD) as appropriate. We have used nonparametric statistical methods because of the small sample size and ordered nature of the answers of the questionnaire. These methods are suitable for sample size less than 20, ordinal data, and they do not need a normal distribution 10. Wilcoxon-Signed rank test was used to compare the continuous data of two related groups while McNemar test for comparison of categorical data of two related groups. Data were analyzed using PASW Statistics 21, SPSS Inc, USA. A p value of less than 0.05 was accepted as significant.

## Results

Table 3 shows the demography of the participants. Majority were from Saudi Arabia, and Kuwait. Eleven (64.7%) were males. Majority were urologists and 7 were Obstetricians and Gynecologists. Eleven were consultants (64.7%) and 6 were specialists (35.3). Nine attended previous relevant courses (52.9%).

**Table 3:** Demography of the participants

	Number (*N = 17*)	Percent
**Specialty:**		
Urology	10	58.8
Gynecology	5	29.4
Obs & Gyn	2	11.8
**Clinical level:**		
Consultant	11	64.7 %
Specialist	6	35.3 %
**Highest medical degree:**		
MD	4	23.5 %
Jordanian Board	2	11.8 %
FRCS	1	5.8 %
FROG	1	5.8 %
FRCOG	1	5.8 %
Master degree	1	5.8 %
Canadian Board	1	5.8 %
MRCOG	1	5.8 %
DRCOG	1	5.8 %
Fellowship	1	5.8 %
**Gender:**		
Male	11	64.7 %
Female	6	35.3%
**Did you attend previous training on** **female SUI and POP?**		
Yes	9	52.9 %
No	8	47.1 %
**Practice:**		
Governmental hospital	11	64.7 %
Private practice	4	23.5 %
Both	2	11.8 %
**Country**		
Saudi Arabia	5	29.4 %
Kuwait	3	17.6 %
UAE	2	11.8 %
Iraq	2	11.8 %
Bahrain	1	5.8 %
Jordan	1	5.8 %
Egypt	1	5.8 %

All variables are expressed as number and percentage from the total number of participants.

**Figure 2:**
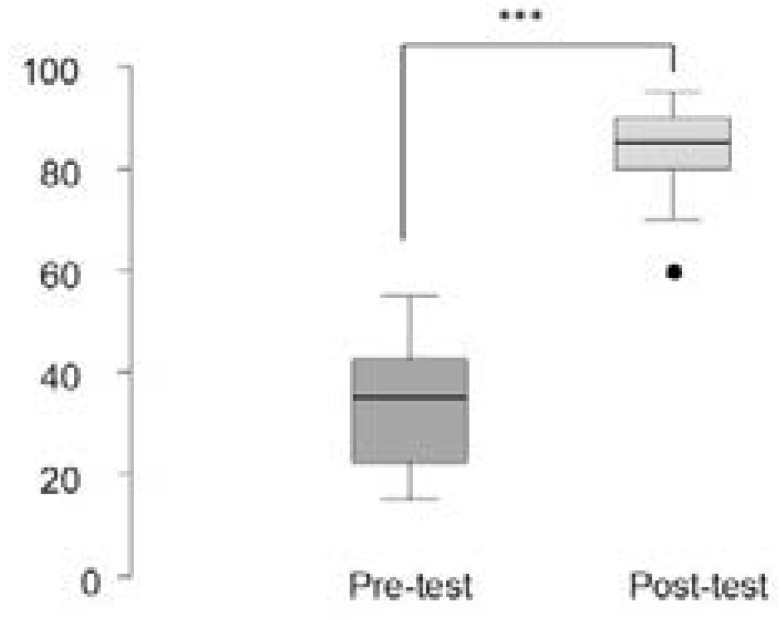
Box-and-whiskers plot of Pre-and Post-course multiple choice question (MCQ) scores before and after the course (n=15). The box represents the 25th to the 75th percentile IQR. The horizontal line within each box represents the median. *** P <0.01, Wilcoxon Signed Rank test.

All participants filled the course evaluation. Fifteen out of 17 candidates completed the pre-and post-test MCQs. One of them passed the pertest MCQs while all 15 participants passed the post-test MCQs (P< 0.001). There was a statistically significant improvement in the post-test MCQ marks compared with the pre-test marks (median (IQ range) 86 (70-95) compared with 35 (15-55); p <0.01) (Figure 2).

Table 4 shows the perception of the participants regarding the course. Encouragement, quality of supervision, organization, and new knowledge learned were highly ranked while case discussions and use of audio-visual methods are areas that need improvement. The mean score for overall assessment of the course was 6.7 out of 7.

**Table 4:** Perception of the participants regarding the course

Attribute	Median (range)	Mean (SD)
Organization of the workshop	7.0 (4 – 7)	6.2 (1.25)
Objectives were met	7.0 (4 – 7)	6.5 (1.01)
Workshop was relevant to my needs	7.0 (5 – 7)	6.6 (0.81)
New knowledge learned	7.0 (4 – 7)	6.6 (0.96)
Usefulness of the workshop	7.0 (4 – 7)	6.4 (1.12)
Talks given by the speakers	7.0 (4 – 7)	6.2 (1.11)
Use of audio-visual methods	7.0 (4 – 7)	6.0 (1.27)
Value of practical exercises given	7.0 (4 – 7)	6.5 (1.01)
Case discussions were	7.0 (4 – 7)	6.0 (1.21)
Encouraged me to use new techniques in my practice	7.0 (5 – 7)	6.7 (0.79)
Supervision of instructors in practical session	7.0 (4 – 7)	6.7 (0.85)
New surgical techniques learned	7.0 (4 – 7)	6.6 (0.94)
The value of using cadavers is training was	7.0 (5 – 7)	6.5 (0.89)
My overall rating of this workshop is	7.0 (5 – 7)	6.7 (0.79)

The participants' open-ended positive comments were as follows: excellent lectures and speakers, very polite trainers with humble attitude and great experience, good hands-on training, new procedures and techniques learned, excellent and practical training. They also gave the following suggestions to improve the workshop: more practical training, reducing the number of participants on each station, more training on genital prolapse surgery, and hands-on training in the hospital,

## Discussion

To our knowledge, our structured urogynecology cadaver-based educational hands-on course is the first of its kind in the literature. This course was highly valued by the participants who came from all over the Middle East. The course was enjoyable and achieved its objectives while the participants gained new skills. Their prior knowledge on female urinary incontinence and pelvic floor prolapse was weak although half of them reported attending previous relevant courses. The knowledge on the topics of interest significantly improved after the course. MCQs are objective, reliable, and valid for assessing knowledge gained by educational programs 5, 11.

Live operating theatres should not be the place to start learning surgical skills but rather to consolidate them. Gynecologist and urologist should reach competency before operating on live patients. This may be achieved by training on human cadaver which allows performing surgery in a real-life setting without stress or fear of causing injuries. Small groups allow individual candidates to have a good opportunity to be trained on specific surgical tasks. This was reflected on the highly positive evaluation of our course by the participants. Previous urogynecology cadaver workshops have focused solely on understanding anatomy while it was used to improve surgical skills in other specialties. Levine et al 2009 reported their experience in laparoscopic cadaver workshop which improved the surgical skills^12^. Because of the tissue fixation, cadavers lose their texture and consistency which may limit the scope of surgical training 5.

Condous et al found, in a prospective observational study, that inexperienced participants benefit the most from skills training 13. In contrast, Schreuder et al found that all participants at different levels of experience were satisfied by the exercises performed during laparoscopy courses 14. Overall, the participants highly ranked our course. The evaluation of our courses by the participants might be influenced by many factors. Less experienced surgeons tend to be polite or feel obligated to highly rank the course in exchange for a chance to be trained 15. They might also give positive responses because they have been exposed to a new experience. Experienced surgeons are usually more critical because to want to refine their skills. Majority of our participants were consultants which is assuring that the evaluation is reliable. This was properly achieved because we planned the course to be tailored according to individual learner's needs. Our study shows that urogynecology cadaver-based hands-on workshops are feasible and can be highly valuable. Using cadavers in our course is possibly another explanation for the high ranking of our course 6.

## Limitations

One of the limitations of our study is that we did not assess the clinical skills gained by the participants in the operating theatre 16, which is the preferred outcome of this educational activity. This is difficult because majority of the participants are not from UAE and do not have a license to practice in our setting. Majority of participants felt that their operative skills have improved which may encourage them in applying these skills in their clinical practice when they return to their home countries. Inexperienced trainees need supervision till mastering these skills otherwise they will be lost overtime 17–19.

## Conclusions

Gynaecologists and urologists need knowledge and practical skills for less invasive techniques in urogynecology surgery. Our structured urogynecology cadaver-based educational hands-on course was highly valued by the participants who came from all over the Middle East. The course was enjoyable and achieved its objectives while the participants gained new skills.

## List of Abbreviations

MCQs: Multiple Choice Questions
POP: Pelvic Organ Prolapse
SSF: Sacrospinous fixation
TOT: Transobturator VaginaL Tape
UAE: United Arab Emirates
USI: Female Stress Urinary Incontinence

## Declarations

### Data Availability

The de-identified minimal data set for our results will be available if requested.
